# Orbital Cellulitis: A Rare Presentation of Metastatic Bronchial Carcinoma

**DOI:** 10.1155/2011/397451

**Published:** 2011-09-15

**Authors:** Rohit Kumar, Wolfgang Issing

**Affiliations:** Department of Otolaryngology-Head and Neck Surgery, Freeman Hospital, Newcastle upon Tyne, NE7 7DN, UK

## Abstract

*Objective*. We report a rare and unusual case of bronchial carcinoma presenting with symptoms of complications of sinonasal disease. *Case Report*. A 66-year-old lady was referred with a 1-week history of progressive ocular pain, chemosis, and visual disturbance. Computed tomography of the paranasal sinuses revealed frontal and ethmoidal sinus opacification with orbital involvement consistent with a diagnosis of orbital cellulitis secondary to sinusitis. Surgical exploration revealed that the sinuses and right orbit were filled with soft tissue and subsequent histopathological examination of the biopsies indicating metastases from an adenosquamous bronchial carcinoma. Further imaging revealed a large, asymptomatic, bronchial primary with deposits in the brain and liver. The advanced presentation of the disease limited treatment to best supportive care. *Conclusion*. Orbital cellulitis and sinonasal malignancies have a similar pattern of clinical presentation, posing a potential diagnostic pitfall. There are only two previously reported cases of metastatic lung carcinoma in the frontal sinus with 15 cases of sinonasal tract involvement reported overall. There are no reported cases of adenosquamous carcinoma in the sinonasal tract.

## 1. Introduction

We describe a case of a frontal and ethmoidal sinonasal carcinoma secondary to metastatic primary bronchogenic carcinoma, presenting as a referral for orbital cellulitis. There are two reported cases of metastatic lung carcinoma in the frontal sinus [1, 2], with fifteen reported cases to the paranasal sinuses overall [3]. There are no reported cases of adenosquamous carcinoma in the sinonasal tract.

## 2. Case Report

A 66-year-old lady was referred to the Ear, Nose and Throat emergency clinic by ophthalmology with a diagnosis of right-sided orbital cellulitis secondary to sinusitis. The patient gave a one-week history of gradually worsening right ocular pain and periorbital swelling with deterioration in vision. She denied any history of nasal obstruction or discharge. She was a smoker of 20 cigarettes per day and consumed in excess of forty units of alcohol per week. Examination of the eye and orbit revealed significant proptosis with peri-orbital erythema and oedema, chemosis, and diplopia. Colour vision was intact but visual acuity was reduced to 6/12. Nasal examination did not reveal any abnormalities.

**Figure 1:**
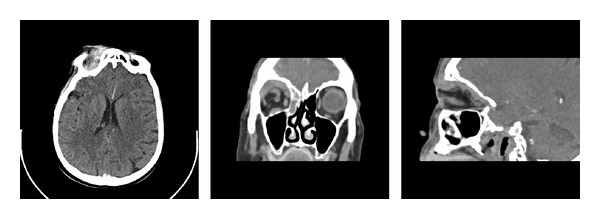
Triplanar views of a noncontrast CT scan showing opacification of the right ethmoidal and frontal sinuses with medial orbital extension.

A CT scan of the paranasal sinuses revealed an area of low attenuation within the right frontal and ethmoidal sinuses with soft tissue inflammation medial to the medial rectus muscle within the orbit. Meningeal enhancement was present along the frontal lobe suspicious of an empyema. In addition, there was a ring-enhancing lesion in the right temporal lobe, again suspicious of infection, but metastatic disease could not be excluded. (Figure 1).

The patient underwent an endoscopic examination of the paranasal sinuses under general anaesthesia. Exploration of the ethmoid bulla and frontal recess revealed a soft tissue mass but no evidence of pus. This tissue also extended into the orbit where it was biopsied via a Lynch-Howarth incision. With the suspicion of sinonasal malignancy being the underlying diagnosis, a staging CT scan was performed with an MRI of the head.

Radiology confirmed the presence of a large right upper lobe lung tumour with hilar lymphadenopathy along with metastases to the brain and liver. Histopathology from the paranasal sinuses and orbit confirmed poorly differentiated adenosquamous carcinoma, suggestive of a bronchial primary. The advanced nature of the disease led to the conclusion that best supportive care would be the mainstay of treatment.

## 3. Discussion

This is rare case of nonsmall cell lung cancer (NSCLC) presenting with late ethmoidal and frontal sinus and orbital metastatic disease. It is an uncommon lung malignancy presenting in an unusual fashion by involving a part of the sinonasal tract that is usually spared with metastatic disease.

Sinonasal malignancies (SNM) account for less than 1 percent of all neoplasms and only 3 per cent of all head and neck cancers [4]. SNMs most commonly involve the maxillary sinus, followed by the nasal cavity, the ethmoid sinuses and the frontal and sphenoid sinuses rarely [5]. They have a male preponderance and mostly occur between the sixth and seventh decades of life. Tumour pathology is most commonly squamous cell carcinoma (SCC) but can include adenocarcinoma, adenoid cystic carcinoma and olfactory neuroblastoma. Metastases to the sinonasal tract are rare with lung, breast, and kidney being common sites of primary origin and with renal cell carcinoma being the most common [6].

Adenosquamous cell carcinoma of the lung is a rare form of nonsmall cell lung cancer (NSCLC) responsible for approximately 3 per cent of all lung cancers [7]. It exhibits histopathological features of both adenocarcinoma and squamous cell carcinoma and has an aggressive potential for rapid growth along with metastatic spread. The most commonly involved distant sites include liver (47%), bone (34%), brain (32%), and adrenals (29%) [8]. Sinonasal involvement from NSCLC is rare with only a handful of isolated cases reported worldwide [1–3].

NSCLC often presents with symptoms related to local disease such as dyspnoea, cough and haemoptysis but can present with signs of distant metastases, most commonly weight loss, pathological fractures, and neurological symptoms associated with central nervous system involvement.

The patient in discussion had no respiratory complaints nor was there a history of previous sinonasal symptoms. The presenting complaints of facial pain and periorbital oedema alongside signs of visual change, chemosis, and proptosis are certainly features of orbital cellulitis; however, the insidious onset is highly suspicious of a malignant cause. The risk factors of heavy smoking and excessive alcohol consumption are further red flag indicators of malignancy.

The findings on any imaging requested must always be interpreted with a close correlation to the clinical picture, and it is, therefore, imperative that the request form is completed with all the relevant history. A ring-enhancing lesion on CT can represent abscess formation; however, it is also a feature of primary tumour or metastatic tumour with central necrosis [9]. 

## 4. Summary

- Metastases from primary lung carcinomas to the paranasal sinuses and orbit are extremely rare with deposits favouring the liver, bone, and brain.
- Renal cell carcinoma is the most common primary malignancy to metastasise to the paranasal sinuses.
- The frontal sinus is the least likely paranasal sinus to be involved in metastatic disease, with the maxillary sinus being the most common.
- Adenosquamous carcinoma is an uncommon type of nonsmall cell lung carcinoma.
- Visual disturbance and ocular pain with objective evidence of orbital compromise should be treated with a high index of suspicion.
- It is imperative to provide radiologists with a clear clinical history to facilitate accurate reporting of requests images.
- Clinicians should be aware of the pitfalls of diagnosis with regards to orbital cellulitis and paranasal sinusitis.

## References

[1] Rombaux P, Hamoir M, Liistro G, Bertrand B (2005). Frontal sinus tumor as the first sign of adenocarcinoma of the lung. *Otolaryngology—Head and Neck Surgery*.

[2] Clarkson JHW, Kirkland PM, Mady S (2002). Bronchogenic metastasis involving the frontal sinus and masquerading as a Pott’s puffy tumour: a diagnostic pitfall. *British Journal of Oral and Maxillofacial Surgery*.

[3] Huang CT, Hong RL (2009). Nasion swelling as the presenting symptom of lung adenocarcinoma. *Journal of Thoracic Oncology*.

[4] Wong R, Kraus DH, Shah JP, Patel SG (2001). Cancer of the nasal cavity and paranasal sinuses. *Cancer of the Head and Neck*.

[5] McMonagle BA, Gleeson M, Gleeson M, Bromley SM, Bouma BE (2008). Nasal cavity and paranasal sinus malignancy. *Scott-Brown’s Otolaryngology, Head and Neck Surgery*.

[6] Luna MA, Barnes L (2001). The Occult Primary and Metastatic Tumors to and from the Head and Neck. *Surgical Pathology of the Head and Neck*.

[7] Shimizu J, Oda M, Hayashi Y, Nonomura A, Watanabe Y (1996). A clinicopathologic study of resected cases of adenosquamous carcinoma of the lung. *Chest*.

[8] Stenbygaard LE, Sorensen JB, Larsen H, Dombernowsky P (1999). Metastatic pattern in non-resectable non-small cell lung cancer. *Acta Oncologica*.

[9] Kendall KA, Senders CW, Gershwin ME, Incaudo G (1996). Orbital and intracranial complications of sinusitis in children and adults. *Diseases of the Sinuses: A Comprehensive Textbook of Diagnosis and Treatment*.

